# Structural Racism is Associated with Assisted Living Location

**DOI:** 10.1093/geroni/igab046.3734

**Published:** 2021-12-17

**Authors:** Lindsey Smith, Paula Carder, Kali Thomas, Robin Baker, Neal Wallace

**Affiliations:** 1 Oregon Health & Science University - Portland State University School of Public Health, Portland, Oregon, United States; 2 OHSU-PSU School of Public Health, Portland, Oregon, United States; 3 Brown University, Brown University/Providence, Rhode Island, United States

## Abstract

Our objective was to measure the association between structural racism, a previously unmeasured but theoretically causal factor, and assisted living communities (ALCs) location as fewer ALCs are located in counties with a greater percentage of the population reported as Black (PPB). We used a recently developed measure of structural racism—the racial opportunity gap (ROG), which compares the economic mobility of Black and White people who grew up in the same area with parents who had similar incomes. We estimated a multilevel mixed-effects bivariate regression model to examine the factors contributing to the presence of ALC. We relied on state and county random effects. The likelihood of an assisted living being located in a census tract in 2019 was significantly positively associated with the percent of the population over the age of 65 (OR=150.1573, p=<0.001), the PPB (OR=2.9916, p=0.004), and higher median incomes (OR=1.0, p=<0.001). In contrast, rurality (OR=0.5656, p=<0.001), unemployment rates (OR=0.0288, p=<0.001), and census tracts that have a high PPB in addition to a high county ROG (OR=.0058, p=0.0137) are all associated with a lesser likelihood of an ALC. The interaction coefficient between the ROG and PPB reverses the previously documented negative association between the PPB and ALC presence. This result empirically supports the premise that structural racism, not population race alone, is a negative determinant of where an ALC is located within a county.

